# Genetic Diversity and DNA Fingerprints of Three Important Aquatic Vegetables by EST-SSR Markers

**DOI:** 10.1038/s41598-019-50569-3

**Published:** 2019-10-01

**Authors:** Xingwen Zheng, Teng Cheng, Liangbo Yang, Jinxing Xu, Jiping Tang, Keqiang Xie, Xinfang Huang, Zhongzhou Bao, Xingfei Zheng, Ying Diao, Yongning You, Zhongli Hu

**Affiliations:** 1 State Key Laboratory of Hybrid Rice, Lotus Engineering Research Center of Hubei Province, College of Life Sciences, Wuhan University, Wuhan, 430072 P.R. China; 2 Guangchang White Lotus Research Institute, Guangchang, Jiangxi 344900 China; 3 Wuhan National Germplasm Repository for Aquatic Vegetables, Wuhan Vegetable Institute of Science, Wuhan, 430065 Hubei P.R. China; 4 Suzhou Institute of Vegetables, Suzhou, 215008 Jiangsu China; 5 School of Forestry and Life Sciences, Chongqing University of Arts and Sciences, Chongqing, 402160 P.R. China

**Keywords:** Plant breeding, Plant molecular biology

## Abstract

Twenty-two sacred lotus (*Nelumbo nucifera*), 46 taros (*Colocasia esculenta*) and 10 arrowheads (*Sagittaria trifolia*) were used as materials and combined with EST-SSR (expressed sequence tag-simple sequence repeats) primers developed by our laboratory. Core primers were screened from a large number of primers that were able to distinguish all materials with a high frequency of polymorphisms. Six pairs, twenty pairs and three pairs of core primers were screened from sacred lotus, taro, and arrowhead, respectively. The SSR fingerprints of these three important aquatic vegetables, producing 17-, 87- and 14-bit binary molecular identity cards, respectively, were separately determined by using the core primers. Since there were few core primers of sacred lotus and arrowhead, 3 and 9 primer pairs with higher polymorphic information content (PIC), respectively, were selected as candidate primers. These core and candidate primers were used to identify the purities of No.36 space lotus, Shandong 8502 taro and Wuhan arrowhead, which were 93.3% (84/90), 98.9% (89/90) and 100.0% (90/90), respectively. The fingerprints, displayed as binary molecular identification cards of three important aquatic vegetables, were obtained, and their purity was successfully determined with EST-SSR labeling technology. Phylogenetic trees were also constructed to analyze the genetic diversity of 22 sacred lotus, 46 taros and 10 arrowheads. This study classifies and identifies germplasm resources and is an important reference to test the authenticity and variety purity of other aquatic vegetables in the future.

## Introduction

With their rich nutrients, fantastic flavor and taste, excellent medicinal value and health care functions, aquatic vegetables have been continuously well received by consumers as important economic crops. There are various kinds of aquatic vegetables, of which *N*. *nucifera*, *C*. *esculenta* and *S*. *trifolia* are important and widely planted in China, Southeast Asia, Australia, and Oceania; these aquatic vegetables have high economic value and a long history of cultivation^1,2^.

SSR fingerprints are inherent in genomes. SSR markers are not affected by external and internal environments, including growth and development time. The advantages of SSR markers are that they are low-cost, fast (within hours), accurate, and reliable. On the one hand, the authenticity of a variety can be effectively identified according to the expected specificity of the DNA fragment. On the other hand, the quality and purity of seeds can be determined by the proportion in the population. Currently, many crop varieties have DNA fingerprints constructed by SSR, such as rice^3^, corn^4^, wheat^5,6^, cotton^7^, and potato^8^. In addition, a set of crop varieties have DNA fingerprints constructed based on EST-SSR markers, such as *Chinese cabbage*^9^, *Cherry*^10^, *Hemarthria sibirica*^11^, *Blueberry*^12^, *Eriobotrya japonica*^13^, and *Asparagus*^14^.

The authenticity of the variety and quality and purity of seeds are extremely significant for crop and vegetable production^15^. However, currently, seed purity identification in China is still dominated by morphological identification^16^. The method of morphological identification is not only time-consuming and expensive but also subject to environmental factors. Therefore, morphological identification cannot quickly and accurately determine purity or variety. However, identification by SSR markers is accurate, can be accomplished within a short time, is low-cost, and is immune to environmental and self factors. Additionally, EST-SSR markers facilitate easy access to base information and metastasis among related species, with a low cost. Thus, EST-SSR markers have vast potential for future development^17^.

Due to the limited research on molecular markers, most aquatic vegetables do not have established molecular marker fingerprints or DNA fingerprints constructed from EST-SSR markers. Furthermore, there is no way to identify the authenticity of varieties or the quality and purity of seeds by molecular markers. Therefore, the purpose of this study is to construct DNA fingerprints for aquatic vegetables (sacred lotus, taro and arrowhead) by using a large number of EST-SSR molecular markers developed in our laboratory and to identify the purity of these aquatic vegetables. We aim to provide theoretical knowledge for the construction of DNA fingerprints and purity identification of other aquatic vegetables.

## Results and Analysis

### Screening of SSR core and candidate primers for three important aquatic vegetables

A total of 11,178 SSR sites were detected by MISA software, and 6,568 pairs of primers were designed by Primer3.0 software. We used a Perl script to synthesize 38 pairs of primers according to electronic polymorphism prediction, and seventy-two pairs of primers were randomly synthesized. Three hundred twenty-five clear bands were obtained by amplifying DNA from 22 scared lotus cultivars with 80 pairs of primers, with an average of 4 bands per pair of primers. From these 80 pairs of primers, 6 pairs of core SSR-labeled primers with polymorphisms that were able to distinguish all 22 materials were selected (Table 1). Three pairs of EST-SSR primers with high PIC (polymorphism information content) were selected as candidate primers for variety purity identification (Table 2). The PIC of each EST-SSR primer pair was calculated using the following formula: ${\rm{PIC}}=1-(\,\mathop{\sum }\limits_{{\rm{i}}=1}^{{\rm{n}}}{{\rm{q}}}_{{\rm{i}}}^{2})-(\mathop{\sum }\limits_{{\rm{i}}=1}^{{\rm{n}}-1}\mathop{\sum }\limits_{{\rm{j}}={\rm{i}}+1}^{{\rm{n}}}2{{\rm{q}}}_{{\rm{i}}}^{2}{{\rm{q}}}_{{\rm{j}}}^{2}\,)$, where *n* is the number of alleles and *q* is the *i*th and *j*th allele frequency^18^.

**Table 1:** Basic information of the six lotus core primers.

Rank	Primers	Repeats	Fragment size range (bp)	Number of polymorphic fragments	The value of PIC
1	NL-61	(AC)6	212–227	2	0.69
2	NL-28	(AT)6	255–261	3	0.67
3	NL-P8	(TTC)6	153–187	3	0.53
4	NL-35	(GT)6	156–162	3	0.74
5	NL-1	(AGTG)6	242–251	3	0.53
6	NL-P9	(AT)7	180–185	3	0.52

**Table 2:** Basic information of the three lotus candidate primers.

Rank	Primers	Repeats	Fragment size range (bp)	Number of polymorphic fragments	The value of PIC
1	NL-60	(AG)9	188–210	2	0.19
2	NL-47	(CT)8	225–280	5	0.74
3	NL-P23	(AT)9	248–275	6	0.77

In total, 5,278 SSR loci were detected by MISA software, and 2,858 pairs of primers were designed by Primer3.0 software. We randomly synthesized 100 pairs of primers. Two hundred thirteen clear bands were obtained by amplifying DNA from 46 taro cultivars with 72 pairs of primers, with an average of 3 bands per pair of primers. From these 72 pairs of primers, 20 pairs of core EST-SSR primers with a polymorphism frequency that could distinguish all 46 taro materials were selected (Table 3).

**Table 3:** Basic information of the twenty taro core primers.

Rank	Primers	Repeats	Fragment size range (bp)	Number of polymorphic fragments	The value of PIC
1	CE-24	(GCT)5	248–275	9	0.80
2	CE-67	(AT)7	152–188	13	0.82
3	CE-8	(GAG)5	273–309	8	0.81
4	CE-19	(TC)8	142–217	7	0.74
5	CE-34	(CT)6	114–142	4	0.67
6	CE-17	(AT)9	193–210	5	0.63
7	CE-98	(CCT)6	206–217	4	0.61
8	CE-84	(TC)6	228–242	4	0.61
9	CE-59	(CGA)5	238–263	3	0.53
10	CE-57	(GGT)5	238–253	4	0.47
11	CE-78	(CTT)6	147–170	2	0.41
12	CE-100	(AG)7	206–231	3	0.40
13	CE-28	(TGC)5	242–273	4	0.36
14	CE-63	(AT)7	105–121	2	0.35
15	CE-2	(CGGTGA)5	180–195	4	0.33
16	CE-37	(CTC)5	276–282	3	0.08
17	CE-45	(CAT)5	191	1	—
18	CE-64	(AT)6	273	1	—
19	CE-27	(GCT)5	105–116	3	0.54
20	CE-47	(GA)7	100–115	3	0.24

A total of 3,861 SSR loci were detected by MISA software, and 2,476 pairs of primers were designed by Primer3.0 software. We randomly synthesized 100 pairs of primers. One hundred ninety-three clear bands were obtained by amplifying DNA from 10 arrowhead cultivars with 78 pairs of primers, with an average of 2.5 bands per pair of primers. From these 78 pairs of primers, 3 pairs of core EST-SSR primers with a polymorphism frequency that could distinguish all 10 arrowhead materials were selected (Table 4). Nine pairs of EST-SSR primers with high PIC were selected as candidate primers for variety purity identification (Table 5). For every aquatic vegetable, at least one figure of polymorphism showed in supplementary file 2.

**Table 4:** Basic information of the three arrowhead core primers.

Rank	Primers	Repeats	Fragment size range (bp)	Number of polymorphic fragments	The value of PIC
1	SS-50	(GGA)5	171–196	7	0.80
2	SS-86	(AGC)5	270–287	5	0.62
3	SS-84	(TA)7	213–220	2	0.33

**Table 5:** Basic information of the nine arrowhead candidate primers.

Rank	Primers	Repeats	Fragment size range (bp)	Number of polymorphic fragments	The value of PIC
1	SS-82	(TC)9	135–160	6	0.84
2	SS-62	(GCTG)5	180–189	4	0.75
3	SS-68	(CGA)5	278–290	5	0.72
4	SS-95	(GGA)6	127–136	3	0.67
5	SS-43	(TC)8	250–263	3	0.65
6	SS-41	(GCC)5	192–201	4	0.63
7	SS-93	(CGC)6	222–235	4	0.63
8	SS-88	(TGC)5	186–198	4	0.58
9	SS-80	(CTC)6	142–153	3	0.57

### DNA Fingerprints of three important aquatic vegetables

According to the sequences of the primer alleles presented in Table 4, the scored results, marked as “0” and “1” for the SSR molecular marker bands of sacred lotus core primers, were counted into binary data as 17-bit binary molecular identity cards of 22 sacred lotus cultivars (Table 6).

**Table 6:** Binary molecular identity of twenty-two lotus varieties.

Rank	Name	Binary molecular identity card	Rank	Name	Binary molecular identity card
1	Cunsan lotus	01100101001101010	12	Wufei lotus	10001110010110011
2	Furong lotus	10100101001001100	13	Zhuoshang lotus	10001100010101010
3	Baixiang lotus	10100101010001100	14	Jiande red flower lotus	10001001010001010
4	Hongxiang lotus	10100101010011100	15	Yixian lotus	10001011010011010
5	Chuzhou white lotus	10010001001001110	16	Qiushui changtian lotus	10001101100101000
6	Tuxuan lotus	10010101001001100	17	Chongtai lotus	10001101010101011
7	White flower jian lotus	10010101001001110	18	Donggua lotus	10001001100001100
8	Red flower jianlotus	01010100001100010	19	No.36 space lotus	10001101010001100
9	Baiye lotus	01001101010001100	20	Riza lotus III-1	10001100010001101
10	Jingguanglotus II	10001101001001100	21	Riza lotus III-2	01001100010001101
11	Star peony	01010100010100010	22	Wuzhi lotus II	01001101001100010

According to the sequences of the primer alleles presented in Table 5, the scored results, marked as “0” and “1” for the SSR molecular marker bands of taro core primers, were counted into binary data as 87-bit binary molecular identity cards of 46 taro cultivars (Table 7).

**Table 7:** Binary molecular identity of forty-six taro varieties.

Rank	Name	Binary molecular identity card
1	Qionglai red-billed taro	010001101000010001000010010000000000001010011111100101010000101111100000011000000110001
2	Xinjin white-billed taro	010011110000010001010010010000001110111011011111001101100000101110010100010000100101001
3	Renshou Wuganqiang taro	010011110000010001010010010000001110111011111111001101100000101110010100010000100101001
4	Pengshan black stem taro	010011110000010001011010010000001100001000011111001010100000101111010000011000000110001
5	Jiange red stem taro	010011110000010001010010010000000000111011011111001101100000101110010100010000100101001
6	Dayi dryland taro	010011101000010001000010010000000010101010011111100011010000101111100000011000000101001
7	Longmen dryland taro	010011110000010001000010110000001110111011111111001101100000101110010100010000100101001
8	Tongji dark stem taro	010011110000010001010010110001001110111011111111001101100000101110010100010000100101001
9	Chengbei Jintang taro	010011110000010001010010110000001110111011011100001101100000101110010100010000100101001
10	Baoning taro	010011110010110010010101000100001101111110011100100101001010100111001011010010001101001
11	Baiqiao Jintang taro	010011110000010001000010110000000000001000011100001101100000101110010100010000100101001
12	Baoning red stem taro	010011110000010001010010110001001110111011111100001101100000101110010100010000100101001
13	Saijin taro	010011101010110001010010010001000000001010011100100011010000101111101000011000000110001
14	Xingwen dryland taro	010011110000010001010010110000001110111011111100001101100000101110010100010000100101001
15	Huilong taro	010011110010010010010101000100001101111110111100100101001010100011001011010010001101001
16	Chengxi cyan stem taro	010011110000010001010010110001001110111011111100001101100000101110010100010000100110001
17	Yongan Wuganqiang taro	010011110010110010010101000100001101111110111100100101001010100111001011010010001101001
18	Dazhu taro	010011110100010001010010110001001110111011111100001101100000101110010100010000100101001
19	Dongxiang red stem taro	010111110100010001010010110001001110111011111100001101100000101110010100010000100101001
20	Taihe red taro	010101110000010001000010110000001110111011111100001101100000101110010100010000100101001
21	Gongping black stem taro	010111110000010001010010110000001110111011111100001101100000100110010100010000100101001
22	Lantian taro	010111110000010001011010010000001100011000111100001010100000101111010000011000000110011
23	Qionglai black stem taro	010111110000010001010010110001001110111011111100001101100000101110010100010000100101001
24	Jintang Wujiaoqing taro	010111110000010001000010110000001110111011111100001101100000101110010100010000100101001
25	Shuangliu wetland taro	010111110010110010010101000100000001111110111100100101001010100011001011010010001101001
26	Yumen Tangbao taro	010111110001011001000010010000001110101000011100100011010001001111010000010000010110001
27	Xinchang betelnut taro	010111101000010001010010010000000010101010011100100011010000101111010000011000000101001
28	Lipu taro	010111110001011001000010010000001110101000011100100011100000101111010000010000010110001
29	Sichuan head taro I	010111110000010001011010010001001100001000111100011010100000101111010000011000000110011
30	Wanxing head taro	010111110000010000011010110001000110101001111100001001001000100110011001010001001101111
31	Lujing big taro	010111110010010100011010110001000110101001111100001001001000100110011001010001001101111
32	Zhongpa head taro	010111110000010001000010010001001110101000011100100011100000100111010000010000010110001
33	Yugan head taro	010111110010010100011010110000000110101001111100001001001000100111010001010001001101111
34	Qingfeng head taro	010111110010010100011010110000000110101001111100001001001000101111001001010001001101111
35	Jiangan Baba taro	010111110100010001110010010001000001111010111100001100100000111111010100110000100110001
36	Dongxiang Goutou taro	010111110100010001110010010001000001111010111100011000100000111111010100110000100110001
37	Ezhou taro	010111110010010010010101000100001101111110111100100101001010100111001011010100001101001
38	WHU greenhouse taro	010011110010010010010101000101001101111110111100100101001010100111001011010010001101001
39	WHU market taro	010011100100010001110010001101110010111110111100100101100000101110000100010000001110001
40	Shandong 8502 taro	010011110100010001110010001101110010111110111100100101100000101110001000000000101100001
41	Zhifang taro	010011110000010001010010001101001110111011011100001101100000101110010100000000100000001
42	WHU private plot taro	010000110010010010010101000100001101111110111100100101001010100111001000000010001101000
43	Paizhong taro	010011110010010010010101000100001101111110111100100101001010100111001000000010001101000
44	Zhuji taro	010011110010010010010101000100001101111110111100100101001010100111001000000010001101001
45	Zuonao Maliuyuan taro	010011110000010001010010001100001110111011111100001101100000100110010100000000100110001
46	Tangxun lake village taro	101011110010010010010000010010001101101110111100100101001010100010011000000000001110000

According to the sequences of the primer alleles presented in Table 6, the scored results, marked as “0” and “1” for the SSR molecular marker bands of arrowhead core primers, were counted into binary data as 14-bit binary molecular identity cards of 10 arrowhead cultivars (Table 8).

**Table 8:** Binary molecular identity of ten arrowhead varieties.

Rank	Name	Binary molecular identity card	Rank	Name	Binary molecular identity card
1	Baoying ziyuan arrowhead	00001100101001	6	Shengdang II arrowhead	00001000010011
2	Gaoyou great white arrowhead	00010010100011	7	Kunming arrowhead	10001000010011
3	Jiangxing produce shendang arrowhead	00001000110011	8	Guangxi sand arrowhead	00001001010001
4	Suzhou yellow arrowhead	00001000110001	9	Guangzhou arrowhead	00001010001001
5	Ziyuan II arrowhead	01000100110011	10	Wuhan arrowhead	00101000010101

### Variety purity identification of three important aquatic vegetables

Among 90 samples of the No.36 space lotus, the bands of 84 samples were regarded as the same characteristic band of the cultivar. The other 6 samples judged to be different cultivars were compared with the characteristic band. Three samples of differential alleles had eight bands, and the other 3 samples had one band. The purity of No.36 space lotus was 93.3% (84/90).

Among 90 samples of Shangdong 8502 taro, the bands of 89 samples were regarded as the same characteristic band of the cultivar. The remaining sample judged to be a different cultivar with 4 differential alleles was compared with the characteristic band. The purity of Shangdong 8502 taro was 98.9% (89/90).

The bands of all 90 Wuhan arrowhead samples were regarded as the same characteristic band of the cultivar, so the 90 samples were identified as the same variety. The purity of Wuhan arrowhead was 100% (90/90).

### Genetic diversity analysis of three important aquatic vegetables

**Figure 1:**
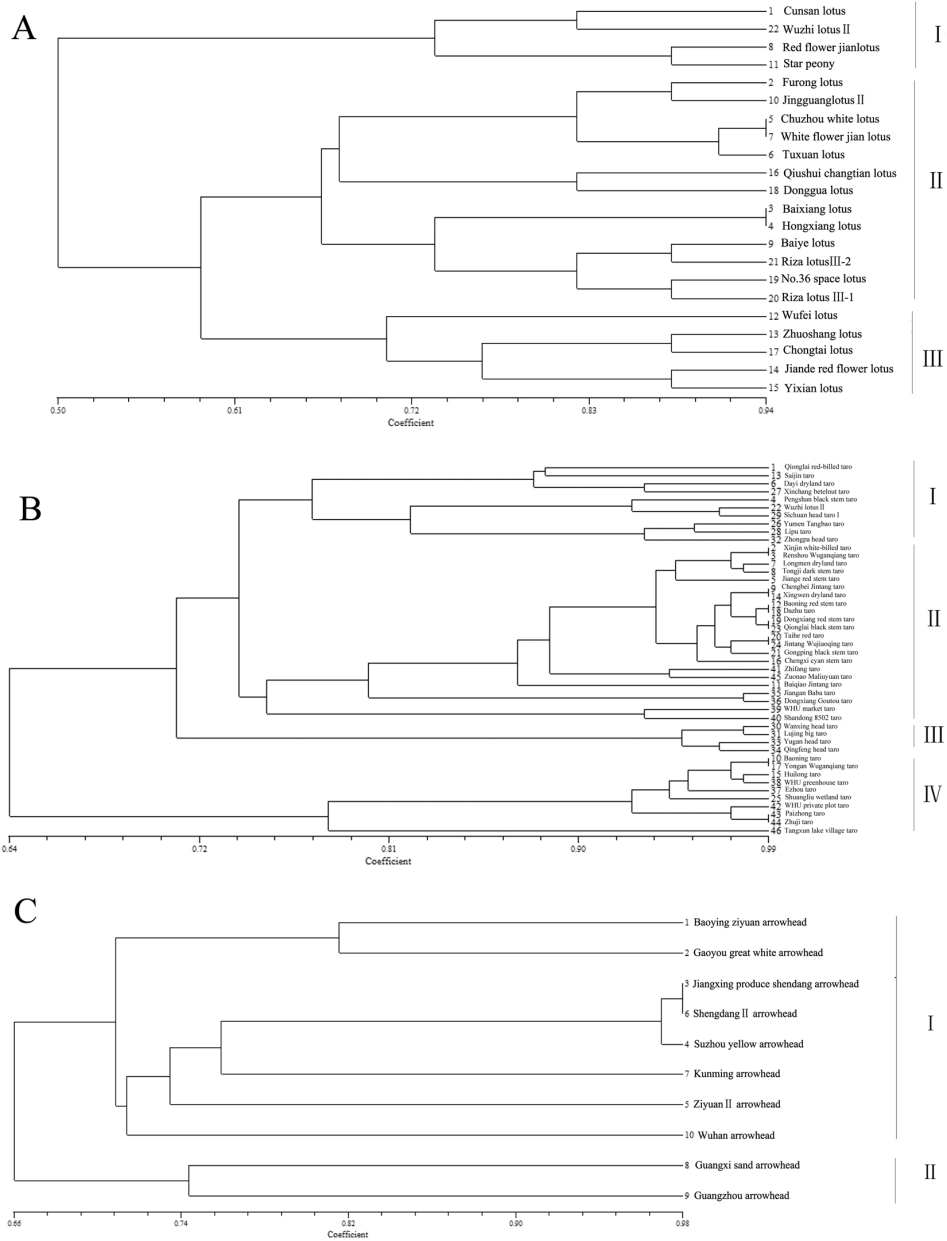
Dendrogram showing the genetic relationships of three important aquatic vegetables. (**A**) Genetic relationships among 22 individual sacred lotus plants based on EST-SSR markers. (**B**) Genetic relationships among 46 individual taros based on EST-SSR markers. (**C**) Genetic relationships among 10 arrowheads based on EST-SSR markers.

A phylogenetic tree of three important aquatic vegetables was constructed (Fig. 1). The Jaccard similarity coefficient ranged from 0.50 to 0.99. Twenty-two individual plants were separated into three major groups, I, II, and III, at a Jaccard similarity coefficient level of 0.65 in sacred lotus (Fig. 1A). The results showed that there was genetic differentiation between lotus with flowers and lotus with roots; lotus with roots was concentrated in group I, while lotus with flowers was mainly concentrated in group III. Forty-six taros were separated into four major groups at a Jaccard similarity coefficient level of 0.74 (Fig. 1B). Two multi-headed taro Jiangan Baba taro and Dongxiang Goutou taro were clustered together, which indicated that there was obvious genetic differentiation between multi-headed and other taro cultivars. Some head taros clustered together in group III, and some clustered together with other types of materials; more than half of the multi-cormels taros were clustered in group II. Arrowhead was separated into two groups (Fig. 1C). A dendrogram with a scale from 0.70 to 0.98 based on Jaccard’s similarity coefficient was constructed and clearly separated the 8 accessions from Jiangsu and Zhejiang provinces into 4 main clusters, while the remaining Guangxi sand arrowhead and Guangzhou arrowhead were classified into group II.

## Discussion

DNA fingerprints are generally constructed by three methods: the characteristic locus method, the single primer method and the core primer combination method, the most efficient of which is the core primer combination method^4,19–22^. Core primers can play important roles in preliminary research, generally leading to a good polymorphism frequency, stability, reproducibility and discriminability. Identifying the the core primers of sacred lotus, taro and arrowhead is a key step for SSR fingerprint construction and purity identification, and it is also a prerequisite for the commercial application of SSR fingerprints. In this study, polymorphic SSR primers were first screened as alternative primers, and a set of SSR primers were found to be the core primers for these germplasm resources, with the smallest number that could completely distinguish all germplasm resources. According to the expansion and complexity of samples and genomes, the number of core primers should be adjusted accordingly. For variety identification, it is better to identify more samples with fewer primers; therefore, the ideal method is to distinguish the most varieties with a single primer. However, this was difficult to achieve, even in arrowhead, the cultivar with the smallest number. As a result, the core primer combination method was adopted in this study to construct fingerprints. In this study, 6 core primers and 3 candidate primers were selected for sacred lotus, 20 core primers for taro, and 3 core primers and 9 candidate primers for arrowhead. Then, 17-, 87- and 14-bit binary molecular identification cards were successfully constructed for 22 sacred lotus cultivars, 46 taro cultivars and 10 arrowhead cultivars, respectively. The DNA fingerprint of sacred lotus was more useful than those of the others^23,24^. The construction of SSR fingerprints for these three important aquatic vegetables is still in its infancy, and in particular, SSR fingerprints for taro and arrowhead have not been reported. SSR fingerprints were constructed by the core primers combination method, which provides useful methodological guidance for the construction of a standard DNA fingerprint database and performing aquatic vegetable mapping analysis in the future.

As a kind of molecular marker with the advantages of high allelic variation, codominance, simple and rapid detection, and good stability, SSRs have been maturely applied in many regions, such as genetic diversity analysis, fingerprint construction, trait marker and genetic linkage map construction. Many agronomists and geneticists have carried out extensive research and application of SSRs^25^. For example, 30 pairs of core primers were used to construct a fingerprint and test the authenticity and purity of Zhongmian Institute 63 cotton. Compared with field trial identification, the results showed that 30 pairs of core primers achieved good identification^26^. From 36 pairs of primers, 3 core primers, MCPI-5, MCPI-16 and MCPI-17, were selected. Identified by MCPI-5 and MCPI-16, the purity of 267 watermelon T-1 hybrids was 97.75%, consistent with the field morphological identification results. Hence, the core primers selected could be used for purity identification of watermelon T-1 hybrids^27^. Other crops, such as rice^28^, rape^29^, potato^30^, maize^31^, eggplant^32,33^, cabbage^34,35^, and soybean^36^, have also been able to be identified.

EST-SSR markers are new molecular markers based on the expression of sequence tag data combined with the characteristics of SSR. These markers combine the dual advantages of SSR and EST and expand the application prospects of SSR. In 2009, EST-SSR markers were applied to the identification of safflower purity, which became the first successful example of the use of EST-SSR markers for purity identification of a crop hybrid population^37^. Subsequently, studies of the purity identification of rice, cabbage, melon (cucumber, melon, watermelon) and other crops have been carried out. The results showed that EST-SSR markers are superior and more sensitive than plant morphology identification in the field, and the purity of detection is generally 2–3% higher than the results from the field^34,38–40^. However, the most commonly used method for the authenticity and purity detection of aquatic vegetables is still morphological identification, which is accomplished by comparison with standard morphological characteristics or determination of morphological characteristic consistency in tested varieties. Due to the limited molecular genetics research, there are a few available SSR markers and no reports of the use of EST-SSR markers to detect the authenticity and purity of aquatic vegetables. Therefore, based on lotus, taro and arrowhead cultivars from all over China, the purity of No.36 space lotus, Shandong 8502 taro and Wuhan arrowhead was determined by EST-SSR and core primer PCR amplification. The purities of these three cultivars were 93.3%, 98.9% and 100%, respectively. It can be seen that these varieties all developed with rigorous breeding and screening. With less genetic variation and higher consistency, the three important aquatic vegetables were close to sacred lotus, taro and arrowhead pure varieties. Because of the genetic differentiation in this cultivar, No.36 space lotus with lower purity is a hybrid cultivar.

In this study, the genetic relationship of a lotus germplasm resources is not related to its geographical origin, as reported in previous reports^41^. At present, lotus can be divided into three types according to its morphological characteristics and its main parts, namely, lotus with seeds, lotus with flowers and lotus with roots^41–43^. The differentiation of lotus with roots and lotus with flowers in this study was consistent with previous research^44^. There is a certain correlation between the genetic relationship and phenotypic traits of lotus. This type of diversity evaluation will provide important reference materials for breeding research of these cultivars in the future. Forty-six taros were separated into four major groups. The main branches were a mixture of materials from different regions and types, and this result indicated that there is no obvious correlation between morphological characteristics or geographic distribution and kinship, possibly due to the cultivar classification of taro based on the variation of its morphological characteristics, which was probably caused by mutations of a few major genes. There is a certain correlation between the regional differentiation of arrowhead cultivars and their genetic differentiation. In Jiangsu and Zhejiang provinces, which belong to the Yangtze River basin, there is a certain differentiation of arrowhead cultivars, and the results of this study suggest that arrowhead is limited by region, especially water limitation. Lower gene exchange led to abundant genetic diversity, which was beneficial to arrowhead breeding. This study can server as a significant reference for the authenticity and purity detection of aquatic vegetable cultivars. Moreover, EST-SSR markers have higher species transferability^45^ and can also be used to study other related species.

## Materials and Methods

### Plant materials

Twenty-two sacred lotus cultivars were used to construct SSR fingerprints, including 14 sacred lotus with seeds cultivars, 7 sacred lotus with flowers cultivars and 1 sacred lotus with root cultivar (Table 9). Forty-six taro cultivated varieties (Table 10) and 10 arrowhead cultivars (Table 11) were used to construct SSR fingerprints.

**Table 9:** Twenty-two lotus varieties used in this study.

Rank	Name	Category	Source	Rank	Name	Category	Source
1	Cunsan lotus**	Lotus with seeds	Xiangtan, Hunan	12	Wufei lotus**	Lotus with flowers	Beijing
2	Furong lotus	Lotus with seeds	Xiangtan, Hunan	13	Zhuoshang lotus	Lotus with flowers	Beijing
3	Baixiang lotus	Lotus with seeds	Xiangtan, Hunan	14	Jiande red flower lotus	Lotus with flowers	Jiande, Zhejiang
4	Hongxiang lotus	Lotus with seeds	Xiangtan, Hunan	15	Yixian lotus	Lotus with flowers	Nanjing, Jiangsu
5	Chuzhou white lotus	Lotus with seeds	Jinhua, Zhejiang	16	Qiushui changtian lotus	Lotus with flowers	Guangchang, Jiangxi
6	Tuxuan lotus	Lotus with seeds	Jinhua, Zhejiang	17	Chongtai lotus	Lotus with flowers	Wuhan, Hubei
7	White flower jian lotus**	Lotus with seeds	Jianning, Fujian	18	Donggua lotus**	Lotus with flowers	Changsha, Hunan
8	Red flower jianlotus	Lotus with seeds	Jianning, Fujian	19	No.36 space lotus*	Lotus with seeds	Guangchang, Jiangxi
9	Baiye lotus	Lotus with seeds	Guangchang, Jiangxi	20	Riza lotus III-1	Lotus with seeds	Wuhan, Hubei
10	Jingguanglotus II	Lotus with seeds	Guangchang, Jiangxi	21	Riza lotus III-2	Lotus with seeds	Wuhan, Hubei
11	Star peony	Lotus with seeds	Guangchang, Jiangxi	22	Wuzhi lotus II**	lotus with root	Wuhan, Hubei

^*^90 materials for purity identification, **Control sample for purity identification.

**Table 10:** Forty-six taro varieties used in this study.

Rank	Name	Category	Area	Rank	Name	Category	Area
1	Qionglai red-billed taro	Taro with multi-cormels	Yangan, Qionglai, Sichuan	24	Jintang Wujiaoqing taro	Taro with multi-cormels	Qingjiang, Jintang, Sichuan
2	Xinjin white-billed taro	Taro with multi-cormels	Wanxing, Xinjin, Sichuan	25	Shuangliu wetland taro	Taro with multi-cormels	Jitian, Shuangliu, Sichuan
3	Renshou Wuganqiang taro	Taro with multi-cormels	Shigao, Renshou, Sichuan	26	Yumen Tangbao taro**	Taro with multi-cormels	Yumen, Yanbian, Sichuan
4	Pengshan black stem taro	Taro with multi-cormels	Pengshan, Sichuan	27	Xinchang betelnut taro	Head taro	Xinchang, Dayi, Sichuan
5	Jiange red stem taro	Taro with multi-cormels	Jiange, Guangyuan, Sichuan	28	Lipu taro	Head taro	Jiangyang, Luzhou, Sichuan
6	Dayi dryland taro **	Taro with multi-cormels	Anren, Dayi, Sichuan	29	Sichuan head taro I	Head taro	Chengdu, Sichuan
7	Longmen dryland taro	Taro with multi-cormels	Longmen, Mianyang, Sichuan	30	Wanxing head taro	Head taro	Wanxing, Xinjin, Sichuan
8	Tongji dark stem taro	Taro with multi-cormels	Tongji, Zhongjiang, Sichuan	31	Lujing big taro	Head taro	Lujing, Yingshan, Sichuan
9	Chengbei Jintang taro	Taro with multi- cormels	Chengbei, Jiange, Sichuan	32	Zhongpa head taro	Head taro	Zhongpa, Yuechi, Sichuan
10	Baoning taro	Taro with multi-cormels	Baoning, Langzhong, Sichuan	33	Yugan head taro**	Head taro	Yugan, Yanbian, Sichuan
11	Baiqiao Jintang taro	Taro with multi-cormels	Baiqiao, Cangxi, Sichuan	34	Qingfeng head taro	Head taro	Qingfeng, Jianyang, Sichuan
12	Baoning red stem taro	Taro with multi-cormels	Baoning, Langzhong, Sichuan	35	Jiangan Baba taro	Multi-headed taro	Jiangan, Yibin, Sichuan
13	Saijin taro	Taro with multi-cormels	Saijin, Yilong, Sichuan	36	Dongxiang Goutou taro**	Multi-headed taro	Dongxiang, Yihan, Sichuan
14	Xingwen dryland taro	Taro with multi-cormels	Xingwen, Bazhou, Sichuan	37	Ezhou taro**	Taro	Ezhou, Hubei
15	Huilong taro	Taro with multi-cormels	Huilong, Yingshan, Sichuan	38	WHU greenhouse taro	Taro	Wuhan University, Hubei
16	Chengxi cyan stem taro	Taro with multi-cormels	Chengxi, Dazhu, Sichuan	39	WHU market taro	Taro	Wuhan University, Hubei
17	Yongan Wuganqiang taro	Taro with multi-cormels	Yongan, Gaoping, Sichuan	40	Shandong 8502 taro *	Taro	Shandong
18	Dazhu taro	Taro with multi-cormels	Dazhu, Daxian, Sichuan	41	Zhifang taro	Taro	Zhifang, Jiangxia, Hubei
19	Dongxiang red stem taro	Taro with multi-cormels	Dongxiang, Xuanhan, Sichuan	42	WHU private plot taro	Taro	Wuhan University, Hubei
20	Taihe red taro	Taro with multi-cormels	Taihe, Shehong, Sichuan	43	Paizhong taro	Taro	Paizhong, Xiantao, Hubei
21	Gongping black stem taro	Taro with multi-cormels	Gongping, Zizhong, Sichuan	44	Zhuji taro	Taro	Zhuji, Xiantao, Hubei
22	Lantian taro	Taro with multi-cormels	Jiangyang, Luzhou, Sichuan	45	Zuonao Maliuyuan taro	Taro	Louhe, Xiantao, Hubei
23	Qionglai black stem taro	Taro with multi-cormels	Qionglai, Sichuan	46	Tangxun lake village taro	Taro	Hongshan, Wuhan, Hubei

*90 materials for purity identification, ** Control sample for purity identification.

**Table 11:** Ten arrowhead varieties used in this study.

Rank	Name	Category	Area
1	Baoying ziyuan arrowhead	Ziyuan arrowhead	Baoying, Jiangsu
2	Gaoyou great white arrowhead**	Great white arrowhead	Gaoyou, Jiangsu
3	Jiangxing produce shendang arrowhead	Shendang arrowhead	Jiangxing, Zhejiang
4	Suzhou yellow arrowhead	Suzhou yellow arrowhead	Suzhou vegetable research institute, Jiangsu
5	Ziyuan II arrowhead**	Ziyuan arrowhead	Suzhou vegetable research institute, Jiangsu
6	Shengdang II arrowhead	Shengdang arrowhead	Suzhou vegetable research institute, Jiangsu
7	Kunming arrowhead**	Kunming arrowhead	Kunming, Yunnan
8	Guangxi sand arrowhead**	Guangxi sand arrowhead	Baipeng, Liujiang, Guangxi
9	Guangzhou arrowhead**	Guangzhou arrowhead	Guangzhou, Guangdong
10	Wuhan arrowhead*	Wuhan arrowhead	Wuhan University, Hubei

*90 materials for purity identification, **Control sample for purity identification.

### Extraction of genomic DNA from three important aquatic vegetables

From the above young leaves of these materials, genomic DNA^46^ was extracted by the modified CTAB method. Among the materials listed above, genomic DNA from the varieties used for purity identification was collected and extracted from the young leaves of 90 plant samples. DNA was extracted by 1.0% agarose gel electrophoresis and stored at −20 °C for later use.

### Primer amplification

SSR marker detection was performed using a Perl program known as MicroSatellite (MISA, http://pgrc.ipk-gatersleben.de/misa) from the *S*. *sagittifolia* transcriptome, *C*. *esculenta* transcriptome and *N*. *nucifera* transcriptome. Using effective EST-SSR primers developed in our laboratory, genomic DNA was amplified by PCR. The primer sequences for sacred lotus and arrowhead are shown in supplementary file 1. Based on the obtained bands, EST-SSR markers were selected as the core primers with a high polymorphism frequency that could distinguish all cultivars. These core primers were employed to amplify each of the 90 samples and 5 control samples of genomic DNA of No.36 space sacred lotus, Shandong 8502 taro, and Wuhan arrowhead. Because of the lack of core primers for sacred lotus and arrowhead, 3 and 9 pairs of candidate primers were added to the samples. The reaction mixture (15 μL) contained 1.5 μL of buffer (10 × dilution), 0.8 μL of MgCl_2_ (20 mM), 0.6 μL of dNTP (10 mM), 0.5 μL of TaqDNA polymerase, 1 μL of forward primer (10 μM), 1 μL of reverse primer (10 μM), 2 μL of template DNA and 7.6 μL of ddH_2_O. The amplification conditions included an initial denaturation step at 94 °C for 5 min, followed by 35 cycles of denaturation at 94 °C for 30 s, and annealing extension at 54–65 °C, elongation at 72 °C for 30 s and a final step at 72 °C for 5 min. Ten microliters of PCR bromophenol blue buffer was added to the PCR products and placed in the gene amplification instrument (BIOER co.,LTD., Hangzhou, China) at 94 °C to degenerate for 5 min, then quickly cooled on ice and tested with a polyacrylamide gel. The silver staining method was used to display the color, and the electrophoresis results were observed on a slide lamp.

### Data statistics and analysis

According to the polyacrylamide electrophoresis results, a band of the same fragment size was recorded as a marker allele and scored as 1, 0 and 2 for a band, no band and a deletion, respectively. The EST-SSR locus allelic variation was counted for sacred lotus, taro and arrowhead, and the statistical data were input into a computer. Data analyses were performed by using the NTSYSpc package version 2.1^47^. The SSR markers were identified as core primers with indicated polymorphisms that could distinguish all cultivars. A set of binary data were obtained from the “0” and “1” data produced by these markers as the binary molecular identity cards of these cultivars.

The characteristic band of a variety is the common characteristic band of most sample individuals. The band of each sample was compared with the characteristic band, and different allele numbers between cultivars (>1) were determined to indicate different varieties, while (=0) was determined to indicate the same variety. P = X/Y × 100%, where X is the number of individuals of the same variety, Y is the total number of individuals identified and P is the value of purity.

## Supplementary information

Supplementary file
