# Supplementary material for: Genetic Diversity and DNA Fingerprints of Three Important Aquatic Vegetables by EST-SSR Markers
Source: Sci Rep. 2019 Oct 1;9:14074. doi: 10.1038/s41598-019-50569-3 (PMC6773842; doi:10.1038/s41598-019-50569-3)
Supplement: Supplementary file 1 — Supplementary file [file 41598_2019_50569_MOESM1_ESM.pdf]

Genetic Diversity and DNA Fingerprints of Three Important Aquatic Vegetables by EST-SSR Markers

Xingwen Zheng[1,2], Teng Cheng[1], Liangbo Yang [2], Jinxing Xu [2], Jiping Tang [2], Keqiang Xie [2], Xinfang Huang[3], Zhongzhou Bao[4], Xingfei Zheng[1], Ying Diao[5], Yongning You[1]*, Zhongli Hu[1]*

[1]State Key Laboratory of Hybrid Rice, Lotus Engineering Research Center of Hubei Province, College of Life Sciences, Wuhan University, Wuhan, 430072, PR China

[2]Guangchang White Lotus Research Institute, Guangchang, Jiangxi, 344900, China

[3]Wuhan National Germplasm Repository for Aquatic Vegetables, Wuhan Vegetable Institute of Science, Wuhan 430065, Hubei, PR China

[4]Suzhou Institute of Vegetables, Suzhou, 215008, Jiangsu, China

[5]School of Forestry and Life Sciences, Chongqing University of Arts and Sciences, Chongqing, 402160, PR China

Corresponding author:

Yongning You & Zhongli Hu

E-mail address: shangruzhenren@163.com（Y. You）& huzhongli@whu.edu.cn (Z. Hu).

Supplementary file 1 Primers used for sacred lotus, taro and arrowhead

Supplementary file 2 Polymorphisms of sacred lotus, taro, and arrowhead. A) Polymorphisms of CE-19 and CE-20 in taros. B) Polymorphisms of SS-50, SS-48 and SS-9 in arrowhead. C) Polymorphisms of NL-1, NL-28, NL-35, NL-47, NL-P8, and NL-P9 in sacred lotus.

Supplementary file 1 Characterization of 41 primer pairs amplification in three aquatic vegetables			
**Primer name**	**Repeats**	**Forward primer(5'-3')**	**Reverse primer(5'-3')**
NL-61	(AC)6	TACATGGCATGGACTCCACA	GTCACCCCTTGGTTGATTTG
NL-28	(AT)6	CATGTGTGCCTAAACATGCC	TGTGCTTCGCTAGCAAAATG
NL-P8	(TTC)6	CTTCGCAGAGTTCATCCACA	TCTTGTCAGGCTAACGCAAG
NL-35	(GT)6	TTGGGAAACAATCAGAAGGG	TGCAAAATAAAAACCTGGGG
NL-1	(AGTG)6	GCTGACTGCTGAGCCTTTCT	AATGCCTGAAACTTCGATGG
NL-P9	(AT)7	AACTGCGTTAGAAGGCAAGG	TTCATGCTCCAAATGCTCTG
NL-60	(AG)9	TCCAGCTAGGGCTTCTGGTA	TTTCTCCCATTCCTCTCCCT
NL-47	(CT)8	ACCGTGGAAGTGGAAAGTTG	AAGTAGCCGACCTGATCGAA
NL-P23	(AT)9	AGAACGTTGATGGGAAGGTG	CCGACTACGATACCGGAAAA
CE-24	(GCT)5	CATGGAAGGAGACCCCTGTA	CCAAGCATTGCAGGGACTAT
CE-67	(AT)7	CGTAGCAAAGTTGGTGTGGA	GGGAAGAGAGAGGAAGCGTT
CE-8	(GAG)5	AAGGGGAATTTGGAGAGACG	GAGGAAGACCCAGGAGAAGG
CE-19	(TC)8	CATATCTGGGTGTTGGGGTC	CTGGCAGCTGATCATCAAGA
CE-34	(CT)6	TGCCAATCATTGTGGTGACT	CACCATTCAACAATGACCCA
CE-17	(AT)9	CCAAGTTCCAAGGTTGGTGT	CAAAGTTTTTGCCCACGAAT
CE-98	(CCT)6	GAGACGAACCGGAGCTGTAG	CTCTCCACCCCAGCATTATC
CE-84	(TC)6	AGATGTGCCTAGCAGTGGCT	GGGTGGACAACCATCAAATC
CE-59	(CGA)5	TCGAAGAAGGAGGTCGAGAA	GCAGCATCAGAAGAGGAACC
CE-57	(GGT)5	AGGATCAATGTGTTGGACCC	GCTCCAATCGTCCTCATCTC
CE-78	(CTT)6	TCTTTCCCATCTTGTCGTCC	AGCGGAAGAGGGAGGAGATA
CE-100	(AG)7	TGCTCGGGAGAAGATTTGAT	ATGGAAAGGGGCTTGCTCT
CE-28	(TGC)5	GTCAGATGCTGGCATGAAGA	CAATGAGGGCCAAGCTAGAG
CE-63	(AT)7	ATTTGGTGGAAAATGGTGGA	TCATTGGAAATTAAAGTGGCA
CE-2	(CGGTGA)5	AGGTTGCTACTACTCCCGCC	CATCCGAATCCTCCTCGTT
CE-37	(CTC)5	CCCTAGCTAGAAAGACCCGC	GGTCTTTACGGAGTCGGACA
CE-45	(CAT)5	CAAGGTTTGAACCGACGAAT	TTTTTGGTGGAAATGAATTTATGTAG
CE-64	(AT)6	CTCTGTCCAGTGAGCTGTGC	GCCATTTACCAATACACAAAAGA
CE-27	(GCT)5	TCCAGGATGGAAAGGACAAG	GACGCCTTTCACCTCTACCA
CE-47	(GA)7	ACACCGAGTACACAGTGGCA	TAGTGACAACGAGCGTCTGC
SS-50	(GGA)5	CTCCACGGCAAAGAAAAGTC	TCCTGCCAATCAATTCCTTC
SS-86	(AGC)5	AGTCACAAAACGGCAGTTCC	CGCCAAAGCATTCATAAGGT
SS-84	(TA)7	CACAAGCTCAGCCAATCTCA	ATAGGCGATTCTGCTGCTGT
SS-82	(TC)9	GGGTGAAGGTTTTCAAGGGT	TCCCCTCCTCCTCTTTCCTA
SS-62	(GCTG)5	GGATGGAGAAGATCTCGCTG	ATTCCCACCTACAGTGCGAC
SS-68	(CGA)5	GTGCGTCCCTATCGACATCT	CATGATCCTCCCTTTCTCCA
SS-95	(GGA)6	CTCGAGTGGGTTTCAAGGAA	CCCCTCACTCAACAATCCAT
SS-43	(TC)8	AACAACCAGTCAGGCCTCTG	ACCGAGAAGAGGAGGAGGAG

SS-41	(GCC)5	AGGAAGGCAATGTGGATGAG	GCTGGGAGAGAGTGACAAGG
SS-93	(CGC)6	TTCATCTGCACCAGCAAGAC	CAGCCGATCTGCATCAACTA
SS-88	(TGC)5	ACAAGAGAACAACCAACCCG	CCTAGATTTGAGATGGGCGA
SS-80	(CTC)6	CGGTCAAACCCACTACCAGT	AACGCCCAGGACCAGTTAGT

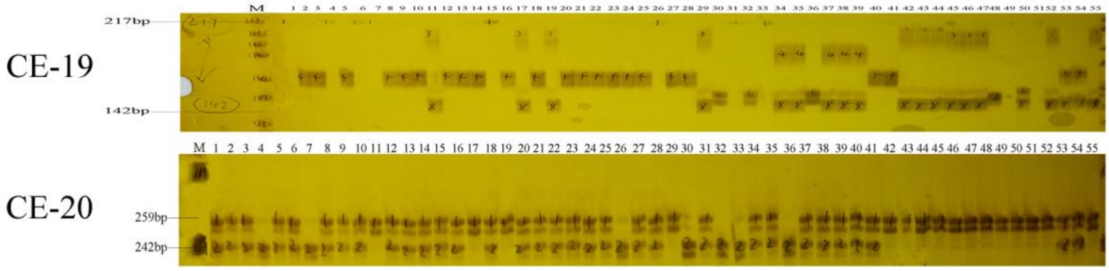
CE-19
217bp
142bp
CE-20
259bp
242bp
M

A

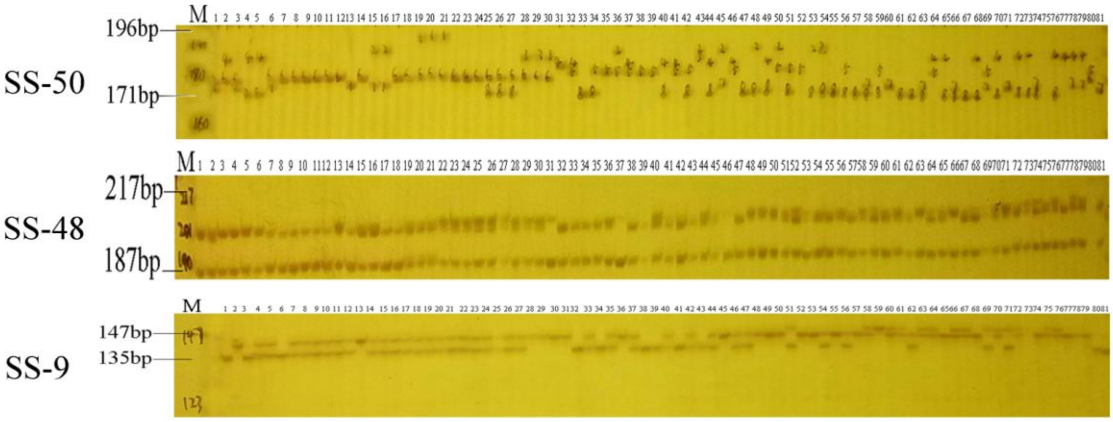
SS-50
196bp
171bp
SS-48
217bp
187bp
SS-9
147bp
135bp
M

B

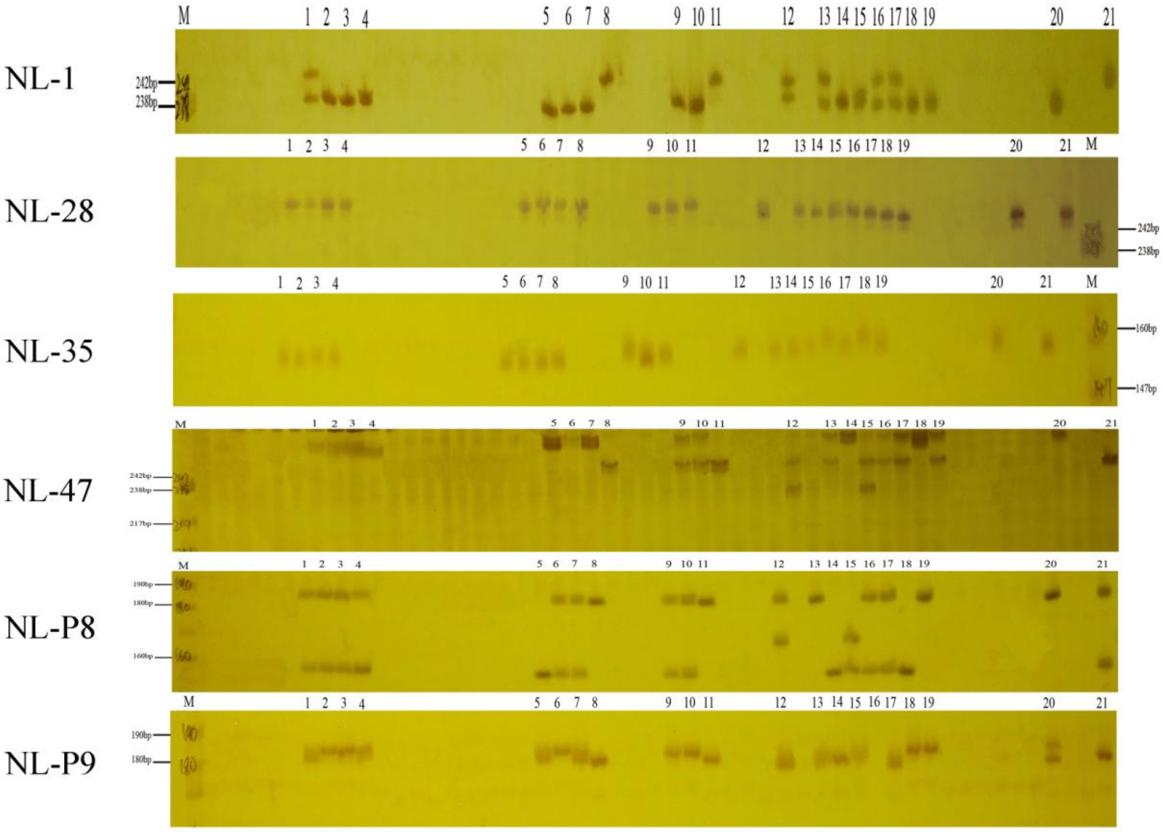
NL-1
242bp
238bp
NL-28
NL-35
160bp
147bp
NL-47
217bp
NL-P8
190bp
180bp
NL-P9
M

C
